# Real‐life experience with non‐vitamin K antagonist oral anticoagulants versus warfarin in patients undergoing elective cardioversion of atrial fibrillation

**DOI:** 10.1111/anec.12766

**Published:** 2020-04-29

**Authors:** Saga Itäinen‐Strömberg, Anna‐Mari Hekkala, Aapo L. Aro, Tuija Vasankari, Kari Eino Juhani Airaksinen, Mika Lehto

**Affiliations:** ^1^ Heart and Lung Center Helsinki University Hospital and University of Helsinki Helsinki Finland; ^2^ The Finnish Heart Association Helsinki Finland; ^3^ Heart Center Turku University Hospital and University of Turku Turku Finland

**Keywords:** anticoagulation, Atrial fibrillation, complications, delays, elective cardioversion

## Abstract

**Background:**

Nonvitamin K antagonist oral anticoagulants (NOACs) are increasingly used in patients with atrial fibrillation (AF) undergoing elective cardioversion (ECV). The aim was to investigate the use of NOACs and warfarin in ECV in a real‐life setting and to assess how the chosen regimen affected the delay to ECV and rate of complications.

**Methods:**

Consecutive AF patients undergoing ECVs in the city hospitals of Helsinki between January 2015 and December 2016 were studied. Data on patient characteristics, delays to cardioversion, anticoagulation treatment, acute (<30 days) complications, and regimen changes within one year were evaluated.

**Results:**

Nine hundred patients (59.2% men; mean age, 68.0 ± 10.0) underwent 992 ECVs, of which 596 (60.0%) were performed using NOACs and 396 (40.0%) using warfarin. The mean CHA_2_DS_2_‐VASc score was 2.5 (±1.6). In patients without previous anticoagulation treatment, NOACs were associated with a shorter mean time to cardioversion than warfarin (51 versus. 68 days, respectively; *p* < .001). Six thromboembolic events (0.6%) occurred: 4 (0.7%) in NOAC‐treated patients and 2 (0.5%) in warfarin‐treated patients. Clinically relevant bleeding events occurred in seven patients (1.8%) receiving warfarin and three patients (0.5%) receiving NOACs. Anticoagulation treatment was altered for 99 patients (11.0%) during the study period, with the majority (88.2%) of changes from warfarin to NOACs.

**Conclusions:**

In this real‐life study, the rates of thromboembolic and bleeding complications were low in AF patients undergoing ECV. Patients receiving NOAC therapy had a shorter time to cardioversion and continued their anticoagulation therapy more often than patients on warfarin.

## 1 INTRODUCTION

Atrial fibrillation (AF) is the most common type of cardiac arrhythmia (Kirchhof et al., 2016). Electrical elective cardioversion (ECV) is an effective way to restore sinus rhythm. However, cardioversion is associated with an increased risk for thromboembolic events such as ischemic stroke and systemic embolism. Without preceding anticoagulation, there is a 5%–7% risk of thromboembolism (Arnold, Mick, Mazurek, Loop, & Trohman, 1992), and with adequate anticoagulation therapy, the risk is reduced to 0.5%–1.6% (Klein et al., 2001). According to the current AF guidelines, adequate oral anticoagulation is recommended for at least 3 weeks before and for a minimum of 4 weeks after ECV in patients with AF > 48 hr or of unknown duration, regardless of their stroke risk profiles (Kirchhof et al., 2016).

Nonvitamin K antagonists (NOACs) have been shown to be as safe and effective as vitamin K antagonists (VKAs) in the setting of cardioversion (Cappato et al., 2014; Flaker et al., 2014; Nagarakanti et al., 2011). Accordingly, many newly diagnosed AF patients initiate NOAC treatment (Forslund, Wettermark, & Hjemdahl, 2016; Huisman et al., 2015). Due to their rapid onset of action, their predictable pharmacokinetics and pharmacodynamics and their ability to be used at fixed doses without routine laboratory monitoring, NOACs offer potential advantages over VKAs and may shorten the waiting times for elective cardioversions (Cappato et al., 2014; Ezekowitz et al., 2018; Goette et al., 2016). At the beginning of warfarin therapy, a labile international normalized ratio (INR) is common, which may prolong the time until ECV can be performed (Ryman, Frick, Frykman, & Rosenqvist, 2003). As an alternative to preprocedural anticoagulation, transesophageal echocardiography (TOE) is useful to exclude cardiac thrombus to facilitate early cardioversion (Kirchhof et al., 2016).

Earlier studies have shown that one in three new NOAC users switches from VKAs (Beyer‐Westendorf et al., 2015; Forslund et al., 2016; Gorst‐Rasmussen et al., 2015). Moreover, one in five patients discontinues NOAC treatment within the first year (Forslund et al., 2016; Gorst‐Rasmussen et al., 2015). Understanding the reasons for the changes between NOACs and VKAs and for the discontinuation of anticoagulation is essential to ensure treatment adherence and to minimize the risk of potential bleeding and thromboembolic events.

The aim of this study was to investigate the reasons for the delays in ECV, the frequency of switching anticoagulants, and the rates of thromboembolic and bleeding events after cardioversion in AF patients undergoing ECV in Helsinki City Hospitals.

## 2 METHODS

This study included consecutive patients undergoing ECV of AF or atrial flutter with NOACs (dabigatran, rivaroxaban, and apixaban) or warfarin at the Haartman and Malmi hospitals of Helsinki city from January 1, 2015, to December 31, 2016. Patients were classified as to whether or not they had or did not have ongoing anticoagulation therapy at the time of AF diagnosis. Data on the patient characteristics, delays to cardioversion, success of cardioversion, anticoagulation treatment, acute (<30 days) complications (stroke or systemic embolism, bleeding events, death or AF relapse), and reasons for changes between warfarin and NOACs as well as changes between different NOACs (<1 year) were collected from electronic case‐report forms.

The duration of AF was evaluated from the documented symptom history in the patient's chart to the time of cardioversion. Delayed cardioversion was defined as postponed or canceled primary ECV, as documented in the medical records by a physician or a nurse. Cardioversion was considered successful if sinus rhythm was restored and maintained for at least five minutes. The primary endpoint measure was stroke or systemic embolism. Patients were classified as having an embolic event if a thromboembolism was confirmed by computerized tomography or magnetic resonance imaging or documented clinically by a physician. The primary safety outcome was any bleeding event reported in the patient's medical record during a 30‐day follow‐up. Major bleeding events were defined according to the International Society of Thrombosis and Haemostasis criteria (Schulman & Kearon, 2005). Switches between NOACs and VKAs and discontinuations of NOAC therapy were analyzed during a 12‐month postcardioversion period.

### 2.1 Statistical analysis

Continuous variables were tested for normality of distribution with the Kolmogorov–Smirnov test. Normally distributed data were analyzed using Student's *t* test and presented as the mean (standard deviation [*SD*]). Skewed continuous data were analyzed using the Mann–Whitney *U* test and presented as the median (interquartile range [IQR]). Categorical variables were compared using the chi‐squared test or Fisher's exact test as appropriate and presented as numbers (%). A *p*‐value <0.05 was considered statistically significant. SPSS Statistics for Windows, version 24.0 (IBM Corp., Armonk, NY, USA), was used for the statistical analyses.

## 3 RESULTS

### 3.1 Study population

A total of 992 ECVs were performed on 900 patients (59.2% men; mean age, 68.0 ± 10.0). The patient characteristics of the NOAC and warfarin groups are shown in Table 1. The mean CHA_2_DS_2_‐VASc score was 2.5 (range 0–8), and 72.3% of the patients had a CHA_2_DS_2_‐VASc score ≥ 2, suggesting a high risk of stroke. The CHA_2_DS_2_‐VASc score was higher in the warfarin group than in the NOAC group (*p* < .001). The index episode was the first manifestation of AF in 540 (60.0%) patients. Transthoracic or transesophageal echocardiograms were performed for 639 (71.0%) and four patients (0.4%), respectively, within 12 months before cardioversion. These revealed one intraventricular thrombus in a patient receiving dabigatran 150 mg ×2. The thrombus was dissolved in a control prior to ECV.

**Table 1:** Patient characteristics

Patients characteristics	NOAC *N* = 596 (60.0%)	Warfarin *N* = 396 (40.0%)	*p*‐value
Age (mean ± *SD*)	67.1 (±10.2)	68.9 (±9.5)	.004
CHA_2_DS_2_‐VASc score	2.2 (±1.5)	2.7 (±1.6)	<.001
HAS‐BLED score	0.8 (±0.7)	1.1 (±0.8)	<.001
Congestive heart failure	61 (11.3)	50 (14.0)	.27
Hypertension	315 (58.1)	257 (71.8)	<.001
Diabetes mellitus^a^	97 (17.9)	76 (21.2)	.52
History of stroke or TIA	13 (2.4)	24 (6.7)	.29
Liver cirrhosis^b^	0 (0.0)	2 (0.6)	.08
Renal failure^c^	13 (2.4)	26 (7.3)	<.001
Alcohol overuse^d^	47 (8.7)	34 (9.5)	.81
Previous myocardial infarction	35 (6.5)	39 (10.9)	.006
Bleeding tendency^e^	4 (0.7)	8 (2.0)	.59
Known thrombophilia	7 (1.3)	7 (2.0)	.36
Antiarrhythmic therapy^f^ precardioversion	61 (10.2)	36 (9.1)	
Antiarrhythmic therapy postcardioversion	95 (15.9)	63 (15.9)	

Abbreviation: TIA, transient ischemic attack.

^a^ Known diagnosis or fasting glucose repeatedly over 7.

^b^ Known diagnosis or Bil > ×2 ULN (upper limit of normal) or ASAT/ALAT > ×3 ULN.

^c^ Creatinine > 140 μmol/L or eGFR < 40 ml/min.

^d^ Women > 14 portions/week and men > 23 portions/week.

^e^ Hemoglobin < 120 mmol/L, thrombocytes < 150 10^f^/L, or previous significant bleeding event.

^f^ Classes I‐IV according to Vaughan Williams classification plus digoxin.

### 3.2 Anticoagulation

Five hundred ninety‐six (60.0%) cardioversions were performed on patients taking NOACs, of which 292 (49.0%) were on rivaroxaban, 162 (27.2%) were on apixaban, and 142 (23.8%) were on dabigatran. Of 396 patients receiving warfarin therapy, 6.1% had a single INR value below 2 during the three weeks preceding ECV. The median number of INR measurements before index ECV was seven (IQR = 5). In total, 99.2% of patients using VKAs and 98.5% of patients using NOACs received treatment for > 4 weeks preceding cardioversion.

**Figure 1:**
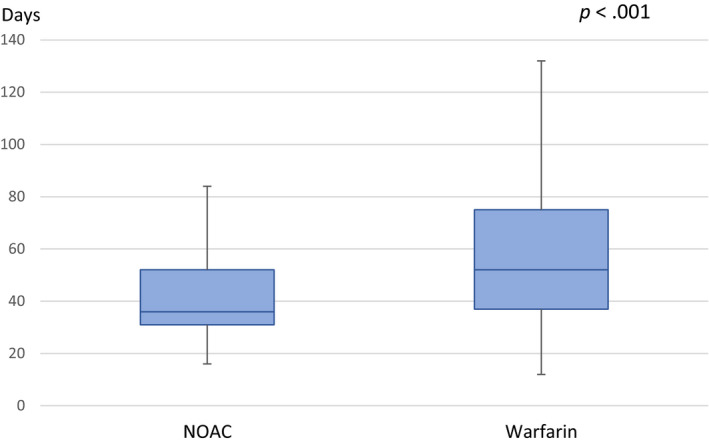
Duration of anticoagulation before elective cardioversion in patients without preexisting anticoagulation therapy. The vertical line through the box represents the median. The upper ends of the boxes represent the upper quartile (3rd quartile), and the lower ends of the boxes represents the lower quartile (1st quartile). The top whisker extends to the maximum value, and the lower whisker extends to the minimum value

In patients with no preexisting oral anticoagulation (556 patients, 61.8%), the mean time from the onset of anticoagulation to ECV in NOAC‐treated patients was 51 ± 41 days, and the median time was 37 days (IQR = 21). In patients who started warfarin treatment, the mean time to cardioversion was 68 ± 42 days, and the median time was 53 days (IQR = 38) (Figure 1). ECV was postponed more often in the VKA group than in the NOAC group (37.4% versus 12.6%, respectively, *p* < .001). The most common reason for visit cancellation was an imbalance of warfarin therapy and labile INR values (Table 2).

**Table 2:** Number (percentage) and reasons for postponing the index cardioversion

Reason	NOAC *N* (%)	Warfarin *N* (%)	*p*‐value
Delay	75 (12.6)	148 (37.4)	<.001
Labile INR values (warfarin)	0 (0.0)	121 (81.8)	
Noncompliance (NOAC)	15 (20.0)	0 (0.0)	
Thromboembolic or bleeding complications	5 (6.7)	1 (0.7)	
Infection	5 (6.7)	1 (0.7)	
Heart failure	9 (12.0)	13 (8.9)	
Hyper‐ or hypothyroidism	3 (4.0)	2 (1.4)	
Travel	10 (13.3)	0 (0.0)	
Surgery	4 (5.3)	0 (0.0)	
Hypokalemia	1 (1.3)	1 (0.7)	
Referral problems	8 (10.7)	2 (1.4)	
Other^a^	10 (13.3)	4 (2.7)	
Unknown delay	6 (8.0)	3 (2.0)	

Abbreviation: INR, international normalized ratio.

^a^ Medication dosage (NOAC) that was too low, diagnostic workup pending, dabigatran kept in administration aids such as dosette boxes prior to ECV.

The majority of cardioversions were successful (89.7% in the NOAC group and 89.1% in the warfarin group; *p* = .75). The success rate was 94.3% for cardioversions with AF duration < 1 week, 93.0% for cardioversions with AF durations of 1 week to 1 month, and 86.3% for cardioversions with AF durations of over 1 month (*p* < .001). The AF recurrence rates during the one 30‐day follow‐up period in the NOAC and warfarin groups were 30.1% (*N* = 179/596) and 28.6% (*N* = 113/396), respectively.

### 3.3 Complications

During the 30‐day follow‐up period after ECV, thromboembolic complications occurred in four patients (0.7%) in the NOAC group and two patients (0.5%) in the warfarin group (*p* = .25). All patients who experienced a stroke or TIA had adequate anticoagulation therapy for at least 3 weeks preceding the cardioversion, and both patients in the warfarin group had an INR value > 2 when thromboembolic complications occurred. Bleeding events that were clinically relevant but not major occurred in 7 (1.8%) patients receiving warfarin and 3 patients (0.5%) receiving NOACs (*p* = .27). The patient characteristics of those with thromboembolic and bleeding complications are shown in Table 3.

**Table 3:** Characteristics of the patients with thromboembolic or bleeding complications

Patient	Complication	Day	Age	Sex	CHA_2_DS_2_‐VASc score	HAS‐BLED score	Anticoagulation
1	Stroke	4	64	Female	1	0	Rivaroxaban 20 mgx1
2	Stroke	2	88	Female	3	1	Warfarin
3	TIA	9	75	Female	3	1	Warfarin
4	TIA	14	61	Male	2	0	Apixaban 5 mgx2
5	TIA	21	78	Male	3	2	Rivaroxaban 15 mgx1
6	Central retinal vein thrombosis	24	79	Male	3	1	Rivaroxaban 20 mgx1
1	GI‐bleeding	30	80	Female	3	2	Warfarin
2	GI‐bleeding	14	60	Male	1	0	Warfarin
3	GI‐bleeding	28	74	Female	5	2	Warfarin
4	GI‐bleeding	13	78	Female	3	2	Warfarin
5	GI‐bleeding	6	68	Male	7	3	Warfarin
6	GI‐bleeding	2	86	Male	5	2	Rivaroxaban 15 mgx1
7	Nosebleed	18	72	Male	1	1	Rivaroxaban 15 mgx1
8	Postmenopausal bleeding	12	79	Female	3	1	Dabigatran 150 mgx2
9	Bleeding unspecified	30	84	Male	4	2	Warfarin
10	Bleeding unspecified	24	71	Male	4	3	Warfarin

Abbreviations: GI, gastrointestinal; TIA, transient ischemic attack.

### 3.4 Anticoagulation after cardioversion

Among 900 patients, 99 patients (11.0%) experienced treatment changes during a 12‐month postcardioversion period: 82 patients switched from warfarin to NOAC, seven patients switched from NOAC to warfarin, and three patients switched between NOACs. The reasons for the changes between OACs are shown in Table 4. One hundred fifty‐seven patients (17.4%) stopped their anticoagulation therapy four weeks after ECV because of a CHA_2_DS_2_‐VASc score less than 2.

**Table 4:** Reason for change in 99 patients (11.0%) who changed their anticoagulation treatment during the study

Reason	No. (%)
Thromboembolic or bleeding complication	8 (8.1)
Patient preference	26 (26.3)
Labile INR values	30 (30.3)
Side effects	4 (4.0)
Economic reasons	3 (3.0)
Unknown	28 (28.3)

Abbreviation: INR, international normalized ratio.

## 4 DISCUSSION

The results of this real‐life study showed that NOACs were associated with a shorter time to cardioversion and fewer postponements of scheduled ECVs than warfarin. Furthermore, our experience supports the view that NOACs are at least as safe and effective as warfarin in real‐life patients undergoing ECV.

The real‐life data evaluating the safety and efficacy of ECV in patients receiving NOACs are sparse (Frederiksen et al., 2018; Pallisgaard et al., 2015). In line with previous studies (Cappato et al., 2014; Goette et al., 2016), the precardioversion use of NOACs enabled faster progression to ECV with fewer cancelations of planned ECVs than warfarin. The median time to the index ECV was 37 days, which was shorter than that of patients treated with warfarin in previous studies (Lehto & Kala, 2003). The tendency toward a shorter time to cardioversion in real‐life patients was also noted in two Danish studies, where in one study, the waiting time for cardioversion in the NOAC group was 28 days, and in the other study, 80% of the cardioversions in the NOAC group were performed within 28 days (Frederiksen et al., 2018; Pallisgaard et al., 2015). In our study, the median time to index ECV for NOAC‐treated patients was higher than the median times of those studies, yet cardioversion was deliberately postponed for only 75 patients (12.6%). This finding indicates that there is a need for improvement in our system to achieve cardioversion within 3–4 weeks in AF patients treated with NOAC. Currently, the advantage of the shorter necessary time before ECV with NOACs is not fully applied in our system. Moreover, TOEs were performed for only 4 (0.4%) patients before ECV. Its use may shorten the waiting times for elective cardioversion also in patients having warfarin and should therefore be used more often in our system.

There are limited data on the reasons for the delay to cardioversion. In this study, patients receiving warfarin had significantly more frequent delays to ECV than patients receiving NOACs. For warfarin‐treated patients, the main reasons for ECV postponements were labile INR values. A major disadvantage of warfarin is its narrow therapeutic window, causing easily subtherapeutic INR levels and delays in ECV. Earlier reports (Itainen et al., 2018; Lehto & Kala, 2003) have suggested that a shorter duration from the onset of AF to cardioversion may decrease the risk for AF recurrence. In our study, the AF recurrence rate was approximately 30%, with no difference between the two groups and, in contrast to previous studies, no correlation between a short AF duration and the recurrence rate. Only 15.9% of patients were treated with antiarrhythmic drugs after ECV, which may have resulted in relatively high recurrence rate. However, the success rate was significantly higher in cardioversion with a shorter AF duration and in cardioversions with an AF duration < 30 days, and the success rate was higher than those of earlier studies (Hellman et al., 2018).

In line with previous studies (Lehto & Kala, 2003; Ruff et al., 2014), the incidence of thromboembolic and bleeding complications in real‐life patients undergoing ECV was low in the present study. Thromboembolic complications occurred in two patients (0.5%) in the warfarin group and four patients (0.7%) in the NOAC group. The CHA_2_DS_2_‐VASc score was higher in the warfarin group than in the NOAC group, but the number of thromboembolic events was so low that possible differences between the warfarin and NOAC groups could not be determined. No major or fatal bleeding events were documented during the follow‐up.

In 11.0% of the patients, the anticoagulation treatment was changed during the one‐year follow‐up after the index ECV. The rate for switches between OACs was lower than those of earlier studies (Beyer‐Westendorf et al., 2015; Hellfritzsch et al., 2017), which indicate that more emphasis was placed on achieving high treatment persistence in AF patients with scheduled ECVs. Most of the changes were from warfarin to NOACs, and the most common reasons for treatment changes were labile INR values and patient preference. Therefore, patients who were prescribed NOACs had a higher drug persistence rate within one year after ECV than patients who were prescribed warfarin. Thromboembolic or bleeding complications were rarely the reasons for changing between OACs.

### 4.1 Limitations

A retrospective analysis does not allow characterization of the study cohort as precisely as a prospective trial. We were dependent on the data recorded by the physicians who performed the cardioversions and who were responsible for follow‐up. Moreover, the clinical characteristics of the two study groups were different. In the warfarin group, patients were older and had more often comorbidities, for example, hypertension and renal failure, that could explain a lower drug adherence and a higher complication rate. Additionally, the onset of arrhythmia was based on the beginning of the patient's symptoms in the medical records, making the exact onset difficult to determine. Also, echocardiography was not performed in every patient in the real‐life setting and we could not collect comprehensive data on, for example, left atrial size which might affect AF recurrence rate. Another limitation of our study is the nonexistence of a laboratory test to confirm patient adherence to NOAC therapy, though all patients scheduled for cardioversion of AF are informed about the importance of adherence. Lastly, due to the low number of complications, the power of the study does not allow for a reasonable estimation of differences between the NOAC and warfarin groups.

## 5 CONCLUSION

The use of NOACs seems to be as safe and effective as that of warfarin in the real‐world setting of elective cardioversion. Patients receiving warfarin have significantly more delays in treatment than patients receiving NOACs, prolonging the time to ECV. Furthermore, our results indicate better adherence with NOACs than with warfarin in the daily care of patients.

## CONFLICT OF INTEREST

Mika Lehto: consultant for BMS‐Pfizer‐alliance, Bayer and Boehringer‐Ingelheim and speaker for BMSPfizer‐alliance, Bayer, MSD and Boehringer‐Ingelheim.

## AUTHOR'S CONTRIBUTION

The sponsors of the study had no contribution to the design, analysis, interpretation, or writing of the study. The first author wrote the first draft of the manuscript, and all the authors participated in subsequent revisions and approved the final version of the manuscript.

## ETHICAL APPROVAL

The study was approved by the Turku University ethics committee. Informed consent was not required due to the register‐based nature of the study.
